# Supplementary material for: Isolation and Identification of Pennogenin Tetraglycoside from Cestrum nocturnum (Solanaceae) and Its Antifungal Activity against Fusarium kuroshium, Causal Agent of Fusarium Dieback
Source: Molecules. 2022 Mar 13;27(6):1860. doi: 10.3390/molecules27061860 (PMC8951829; doi:10.3390/molecules27061860)
Supplement: Supplementary file 1 [file molecules-27-01860-s001.zip › molecules-1593340-supplementary.pdf]

# Isolation and identification of pennogenin tetraglycoside from *Cestrum nocturnum* (Solanaceae) and its antifungal activity against *Fusarium kuroshium*, causal agent of Fusarium dieback

**Erika Valencia-Mejía[1,†], Yeli Y. León-Wilchez[1,†], Juan L. Monribot-Villanueva[1], Mónica Ramírez-Vázquez[2,3], Israel Bonilla-Landa[4], José A. Guerrero-Analco[1,*]**

1 Laboratorio de Química de Productos Naturales, Red de Estudios Moleculares Avanzados, Instituto de Ecología A.C. (INECOL)-Clúster Científico y Tecnológico BioMimic®, Carretera Antigua a Coatepec N. 351, 91070, Xalapa, Veracruz, México.
2 Unidad de Microscopía, Red de Estudios Moleculares Avanzados, Instituto de Ecología A.C. (INECOL)-Clúster Científico y Tecnológico BioMimic®, Carretera Antigua a Coatepec N. 351, 91070, Xalapa, Veracruz, México.
3 Facultad de Ciencias, UNAM. Circuito Exterior, Cd. Universitaria, Copilco, Coyoacán, 04510, CDMX, México.
4 Química Orgánica y Resonancia Magnética Nuclear, Red de Estudios Moleculares Avanzados, Instituto de Ecología A.C. (INECOL)-Clúster Científico y Tecnológico BioMimic®, Carretera Antigua a Coatepec N. 351, 91073, Xalapa, Veracruz, México.
* Correspondence: joseantonio.guerrero@inecol.mx (J.A.G-A); Tel.: +52-228-8421-800 (ext. 3514)
† Authors contributed equally to this work.

**Figure S1.** Mycelial growth inhibition displayed by primary fractions of *C. nocturnum* against *F. solani* at 2 mg mL$^{-1}$
**Figure S2.** Mycelial growth inhibition displayed by secondary fractions from ECn-F4 (2 mg mL$^{-1}$) of *C. nocturnum* against *F. solani*.
**Figure S3.** Mycelial growth inhibition displayed by secondary fractions from ECn-F4 (2 mg mL$^{-1}$) of *C. nocturnum* against *F. kuroshium*
**Figure S4.** Mycelial growth inhibition displayed by secondary fractions from ECn-F4 (1, 0.5 and 0.2 mg mL$^{-1}$) of *C. nocturnum* against *. kuroshium*.
**Figure S5.** $^{1}$H NMR (500 MHz, Py-d5, 50 °C) of pennogenin tetraglycoside
**Figure S6.** Expansion from 3.0 to 7.0 ppm of $^{1}$H NMR (500 MHz, Py-d5, 50 °C) of pennogenin tetraglycoside
**Figure S7.** Expansion from 0 to 3.2 ppm of $^{1}$H NMR (500 MHz, Py-d5, 50 °C) of pennogenin tetraglycoside
**Figure S8.** $^{13}$C NMR (125 MHz, Py-d5, 50 °C) of pennogenin tetraglycoside
**Figure S9**. Expansion from 60 to 115 ppm of $^{13}$C NMR (125 MHz, Py-d5, 50 °C) of pennogenin tetraglycoside
**Figure S10.** Expansion from 0 to 60 ppm of $^{13}$C NMR (125 MHz, Py-d5, 50 °C) of pennogenin tetraglycoside
**Figure S11.** COSY NMR (Py-$d_5$, 50°C) spectra of pennogenin tetraglycoside
**Figure S12.** TOCSY NMR (Py-$d_5$, 50°C) spectra of pennogenin tetraglycoside
**Figure S13.** HSQCed NMR (Py-d5, 50°C) spectra of pennogenin tetraglycoside
**Figure S14.** HMBC NMR (Py-d5, 50°C) spectra of pennogenin tetraglycoside
**Figure S15.** H2BC NMR (Py-d5, 50°C) spectra of pennogenin tetraglycoside
**Figure S16.** Stacked spectra of different TOCSY 1D at different chemical shifts of aglycone.
**Figure S17.** Stacked spectra of different TOCSY 1D at different chemical shifts of glycosidic portion.
**Figure S18.** Stacked spectra of different ROESY 1D at different chemical shifts.
**Table S1.** Optimized dynamic multiple reaction monitoring method (dMRM) parameters for detection and quantification of Phenolic compounds

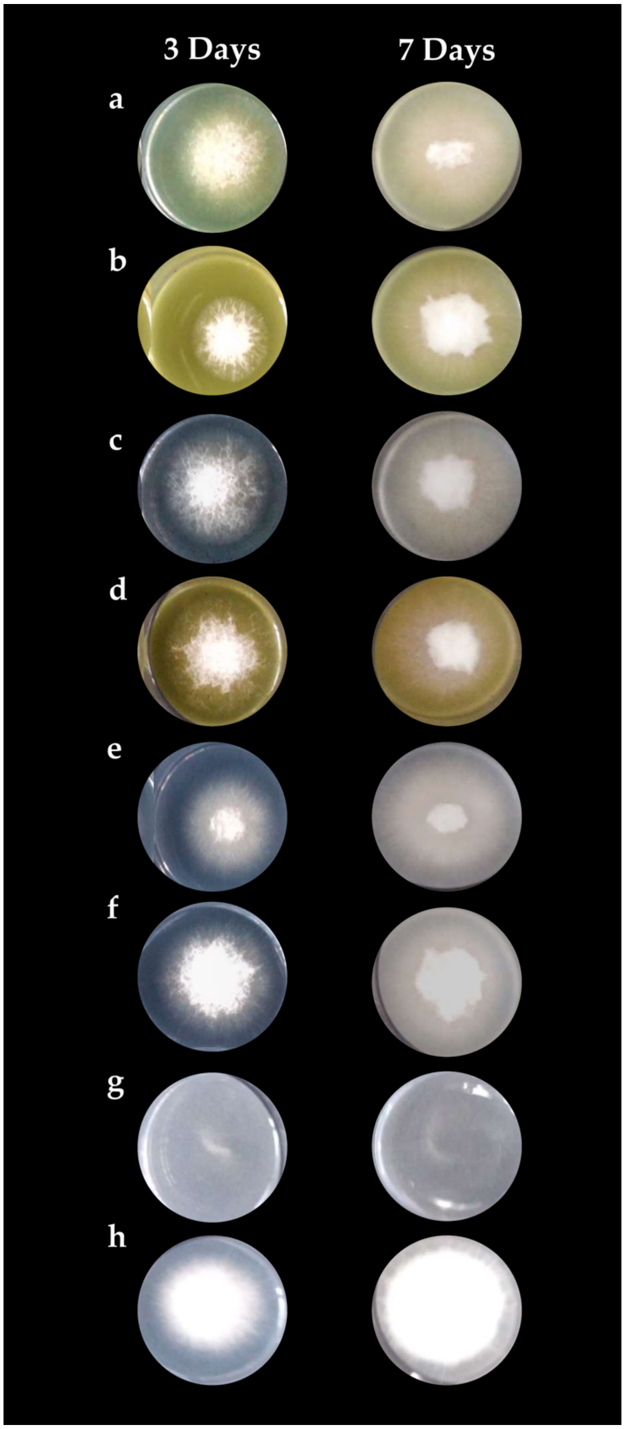


**Figure S1.** Mycelial growth inhibition displayed by primary fractions of *C. nocturnum* against *Fusarium solani* at 2 mg mL$^{-1}$, evaluated three and seven days post-inoculation. **a.** ECn; b. ECn-F1; c. ECn-F2; d. ECn-F3; e. ECn-F4; f. ECn-F5; g. Positive control (C+) thiabendazole 2 mg mL$^{-1}$; h. Negative control (C-) was the vehicle ($H_2O$-MeOH in a 15:85 ratio) where samples were dissolved.

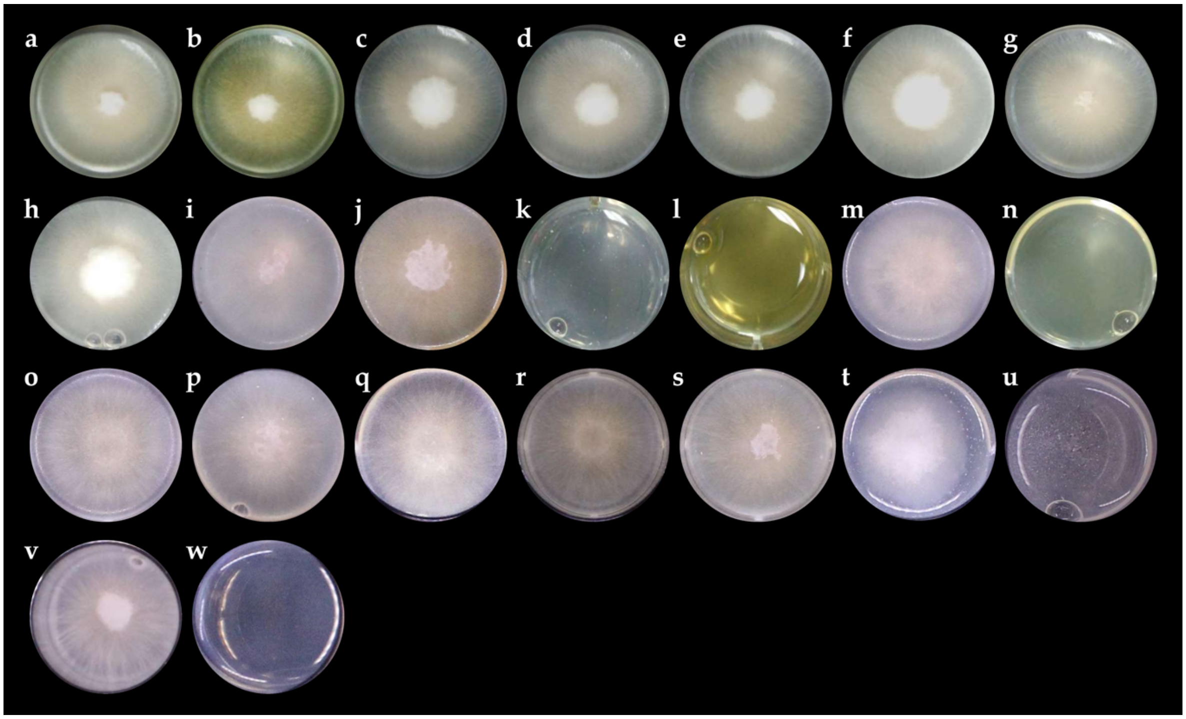


**Figure S2.** Mycelial growth inhibition displayed by secondary fractions from ECn-F4 (2 mg $mL^{-1}$) of *C. nocturnum* against *Fusarium solani*. **a.** ECn-F4-2; **b.** ECn-F4-3; **c.** ECn-F4-5; **d.** ECn-F4-6; **e.** ECn-F4-7; **f.** ECn-F4-8; **g.** ECn-F4-11; **h.** ECn-F4-13; **i.** ECn-F4-14; **j.** ECn-F4-15; **k.** ECn-F4-16; **l.** ECn-F4-17; **m.** ECn-F4-18; **n.** ECn-F4-19; **o.** ECn-F4-20; **p.** ECn-F4-21; **q.** ECn-F4-22; **r.** ECn-F4-23; **s.** ECn-F4-24; **t.** ECn-F4-25; **u.** Positive control (C+) thiabendazole 2 mg $mL^{-1}$; **v.** Negative control (C-) was the vehicle ($H_2O$-MeOH in a 15:85 ratio) where samples were dissolved; **w.** sterility control of the culture medium (PDA).

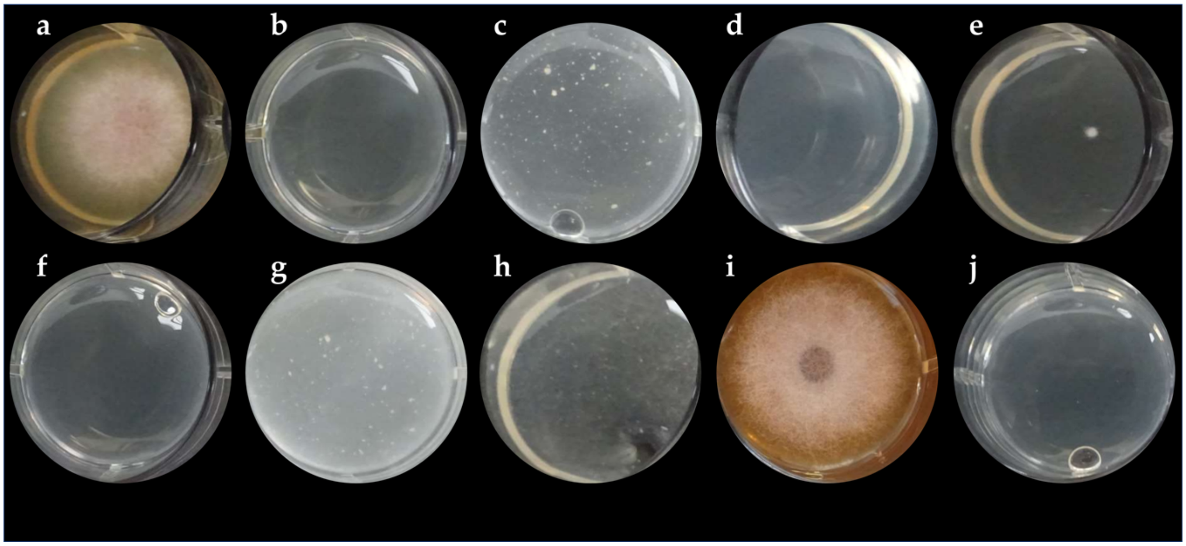


**Figure S3.** Mycelial growth inhibition displayed by secondary fractions from ECn-F4 (2 mg $mL^{-1}$) of *C. nocturnum* against *Fusarium kuroshium*. **a.** ECn-F4-3; **b.** ECn-F4-16; **c.** ECn-F4-17; **d.** ECn-F4-18; **e.** ECn-F4-19; **f.** ECn-F4-20; **g.** ECn-F4-25; **h.** Positive control (C+) thiabendazole 2 mg $mL^{-1}$; **i.** Negative control (C-) was the vehicle ($H_2O$-MeOH in a 15:85 ratio) where samples were dissolved; **j.** sterility control of the culture medium (PDA).

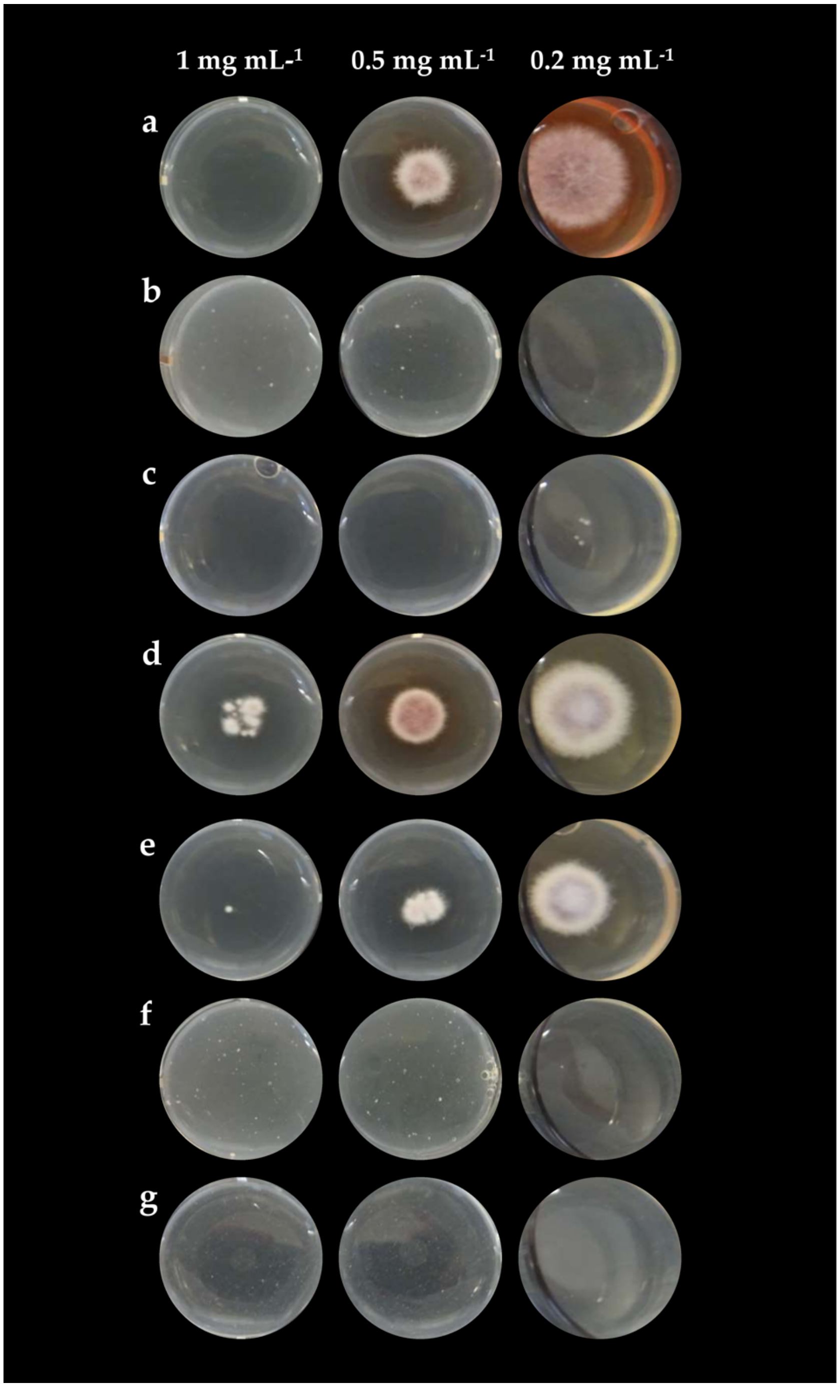


**Figure S4.** Mycelial growth inhibition displayed by secondary fractions from ECn-F4 (1, 0.5 and 0.2 mg $mL^{-1}$) of *C. nocturnum* against *Fusarium kuroshium*. **a.** ECn-F4-16; **b.** ECn-F4-17; **c.** ECn-F4-18; **d.** ECn-F4-19; **e.** ECn-F4-20; **f.** ECn-F4-25; **g.** Positive control (C+) thiabendazole at 1, 0.5 and 0.2 mg $mL^{-1}$.

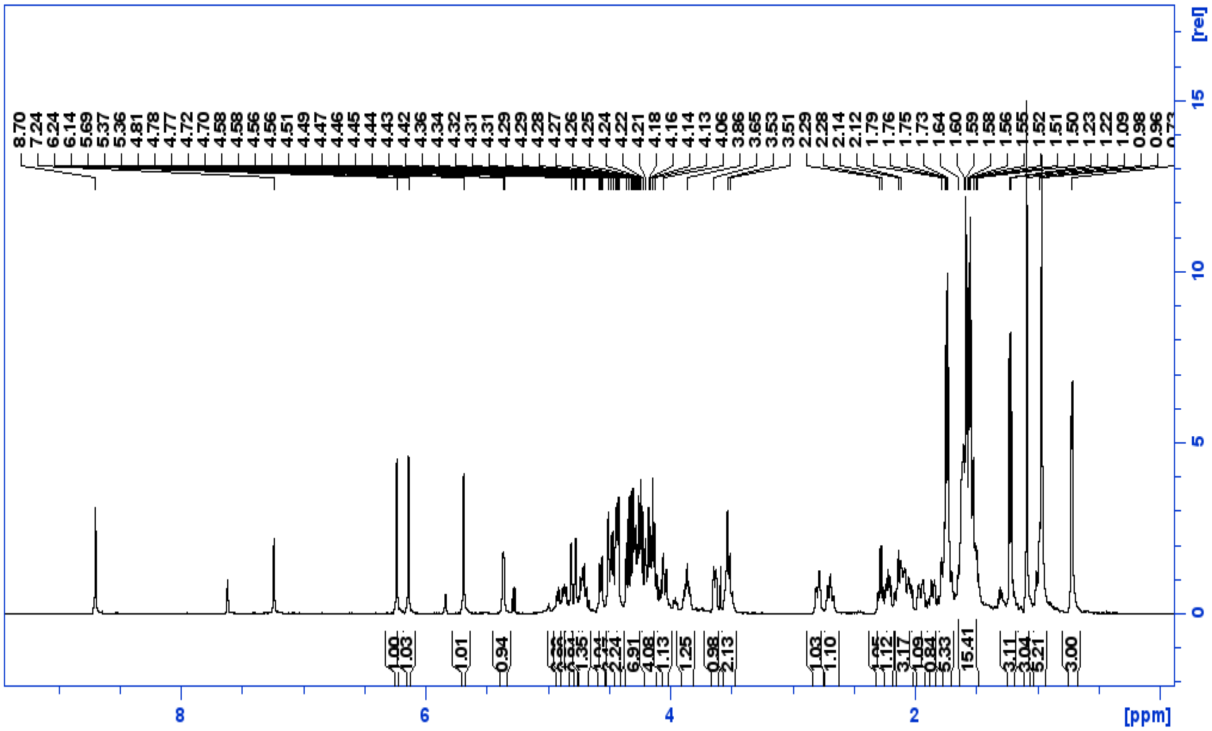

**Figure S5.** $^1$H NMR (500 MHz, Py-$d_5$, 50 °C) of pennogenin tetraglycoside

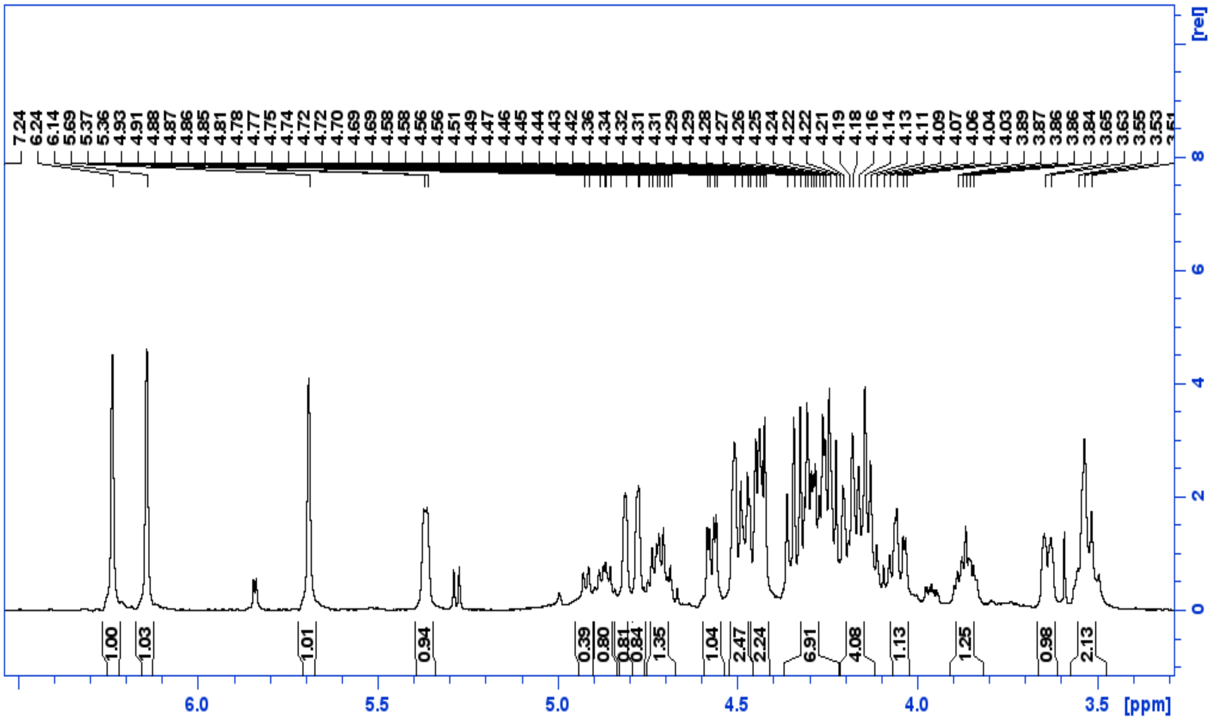

**Figure S6.** Expansion from 3.0 to 7.0 ppm of $^1$H NMR (500 MHz, Py-$d_5$, 50 °C) of pennogenin tetraglycoside

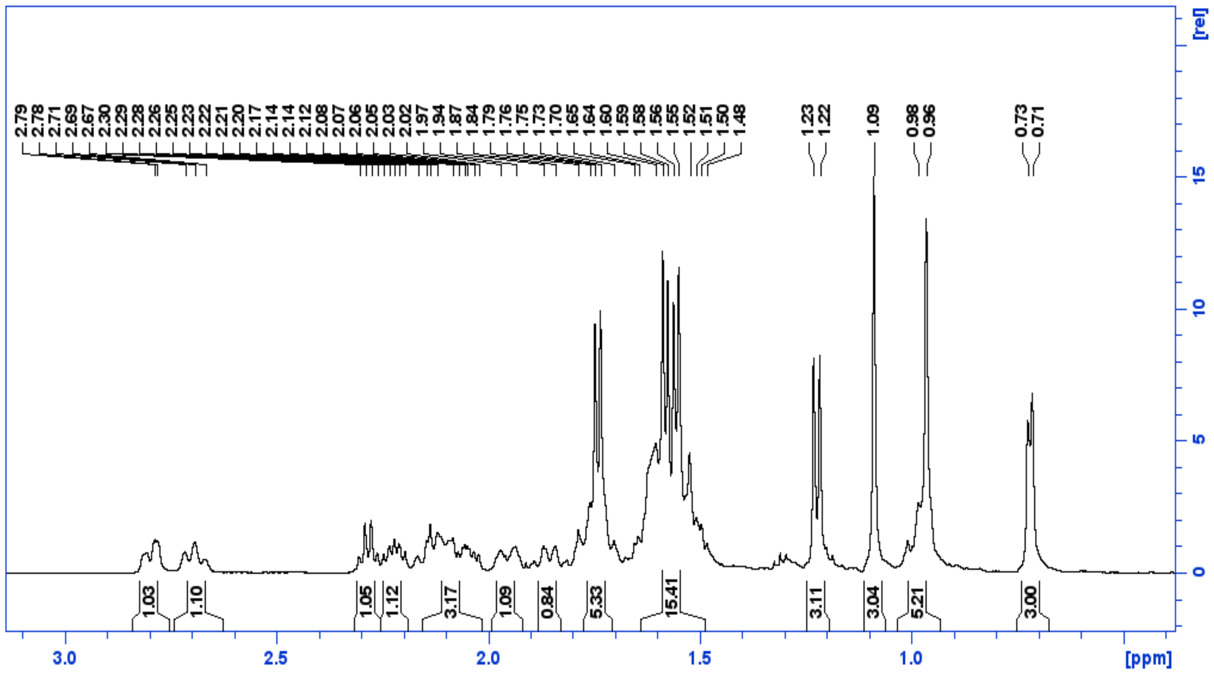

**Figure S7.** Expansion from 0 to 3.2 ppm of $^{1}$H NMR (500 MHz, Py-d5, 50 °C) of pennogenin tetraglycoside

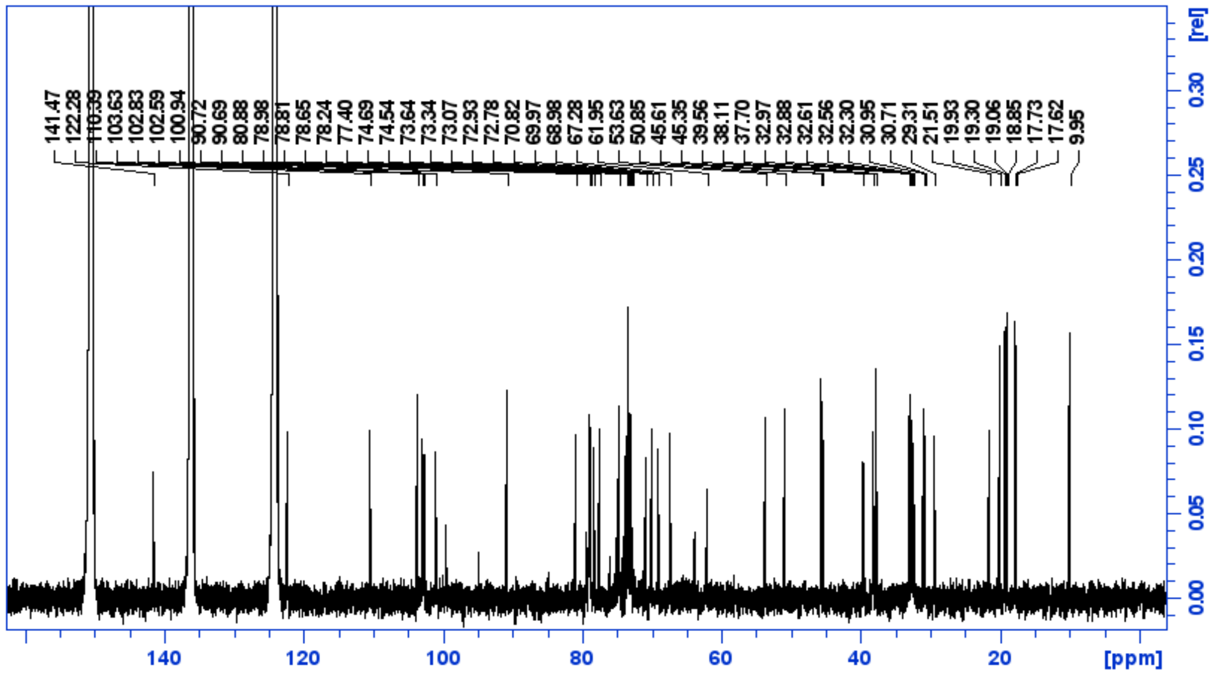

**Figure S8.** $^{13}$C NMR (125 MHz, Py-d5, 50 °C) of pennogenin tetraglycoside

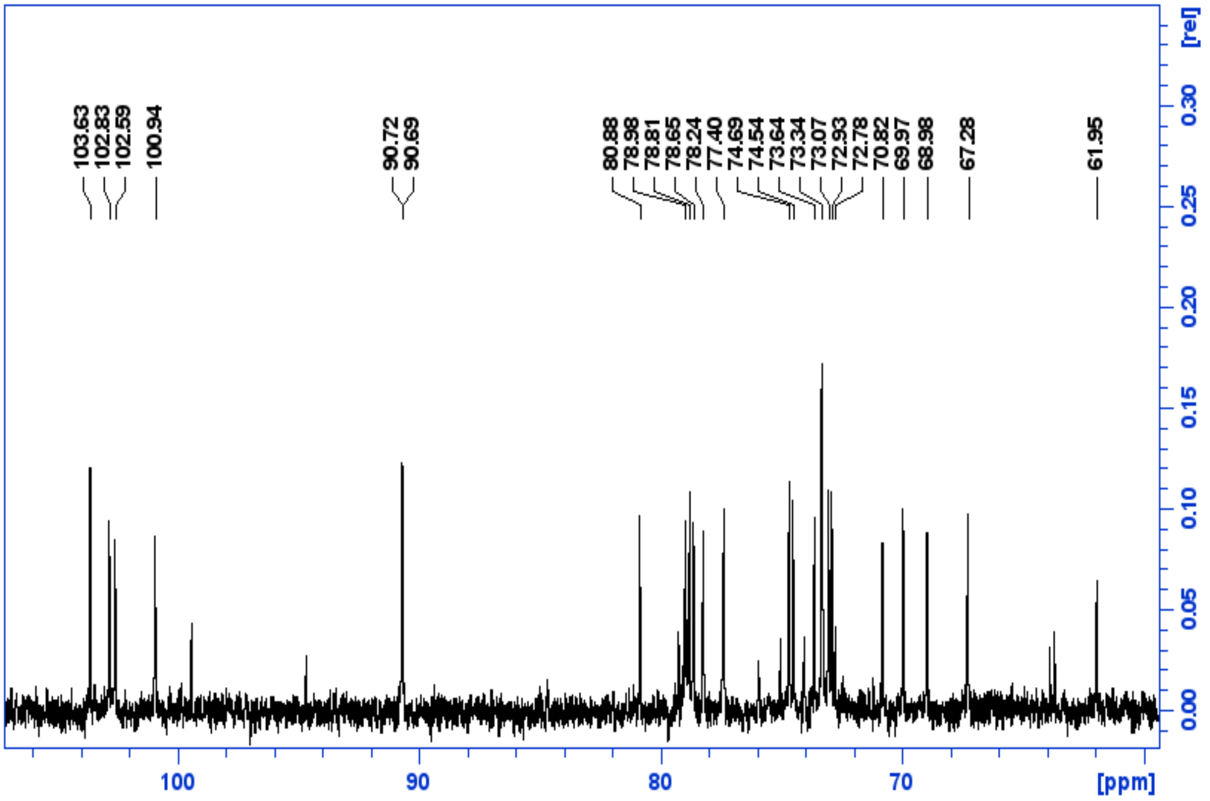

**Figure S9.** Expansion from 60 to 115 ppm of $^{13}C$ NMR (125 MHz, Py-$d_5$, 50 °C) of pennogenin tetraglycoside

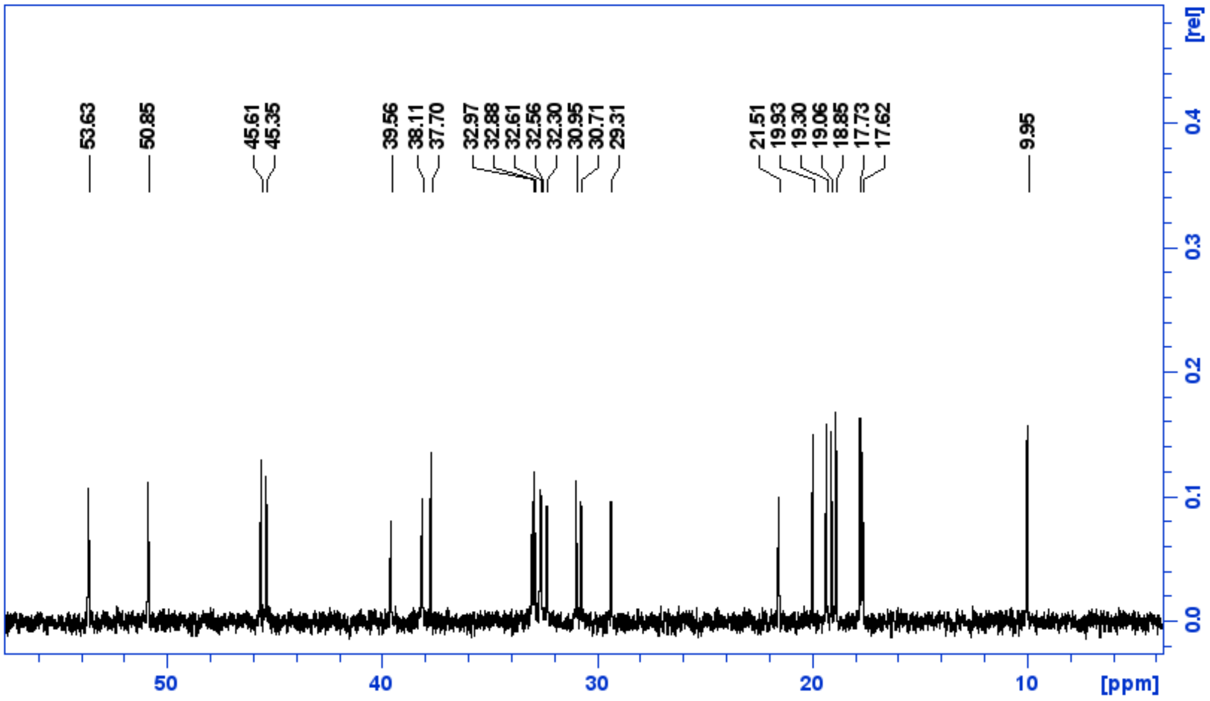

**Figure S10.** Expansion from 0 to 60 ppm of $^{13}C$ NMR (125 MHz, Py-$d_5$, 50 °C) of pennogenin tetraglycoside

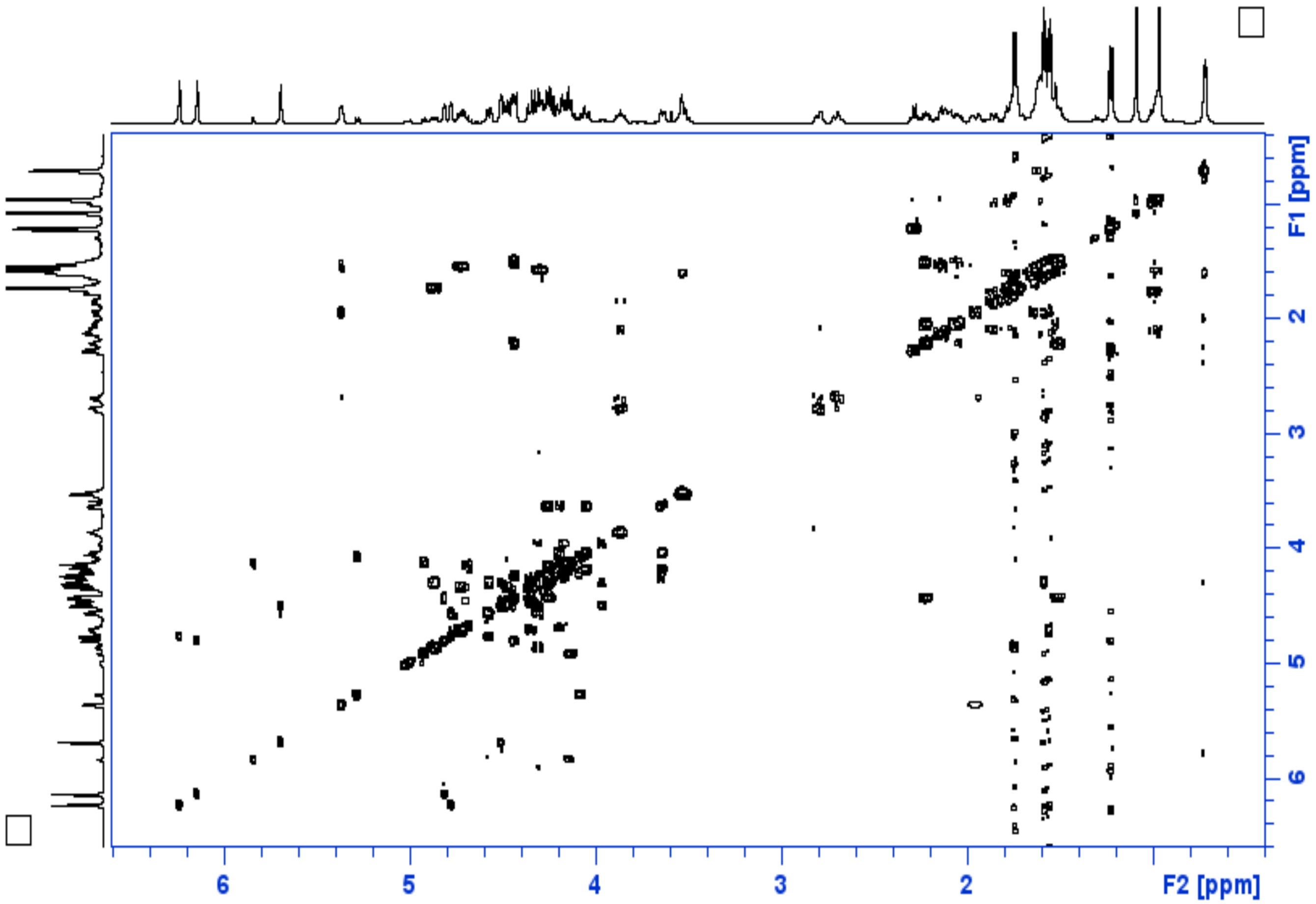


**Figure S11.** COSY NMR (Py-$d_5$, 50°C) spectra of pennogenin tetraglycoside

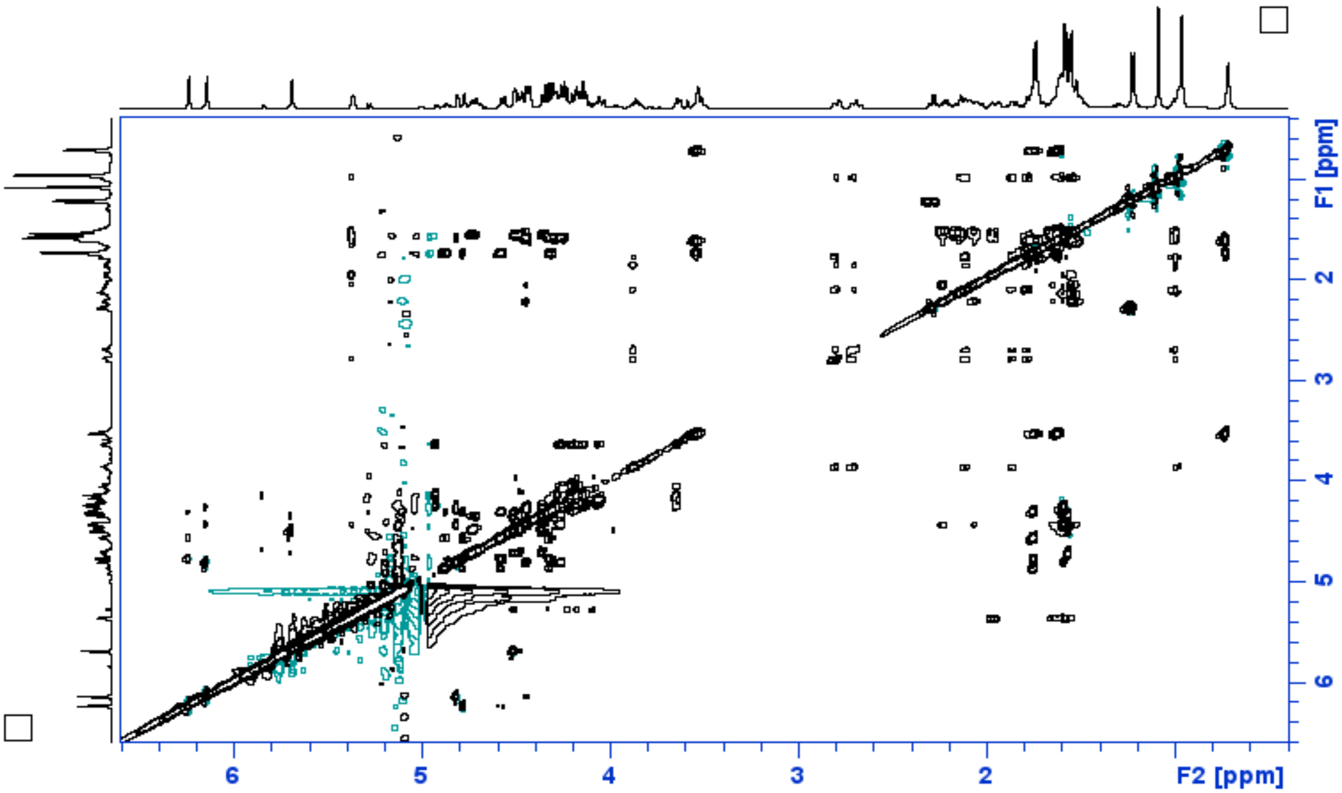


**Figure S12.** TOCSY NMR (Py-d5, 50°C) spectra of pennogenin tetraglycoside

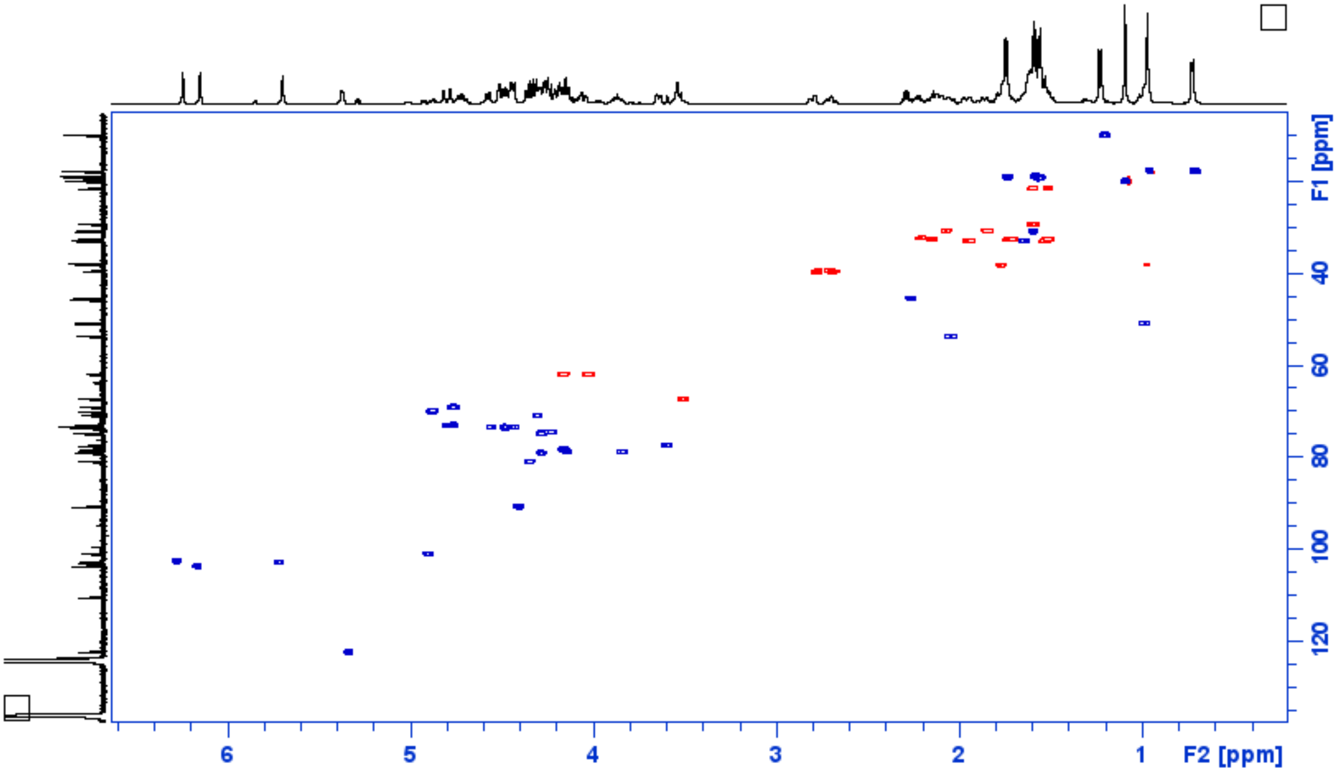


**Figure S13.** HSQCed NMR (Py-d5, 50°C) spectra of pennogenin tetraglycoside, blue cross peaks indicate CH and $CH_3$ and red cross peaks indicate $CH_2$

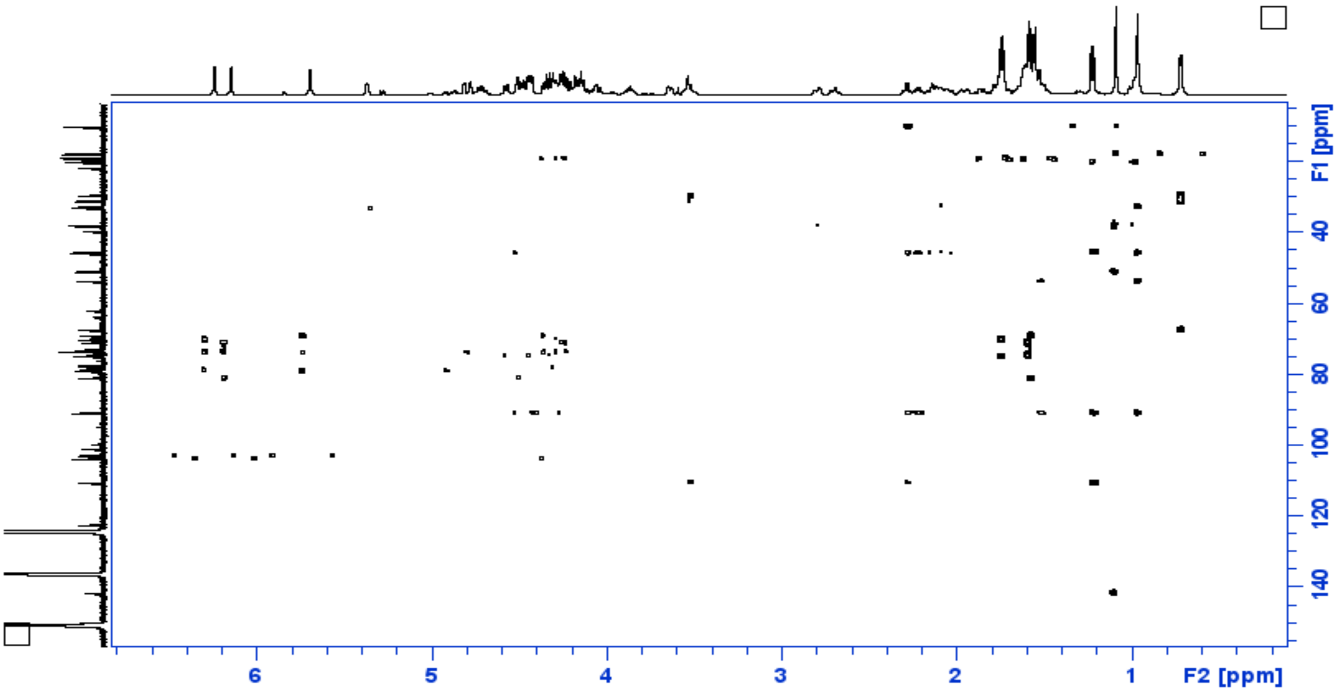


**Figure S14.** HMBC NMR (Py-d5, 50°C) spectra of pennogenin tetraglycoside

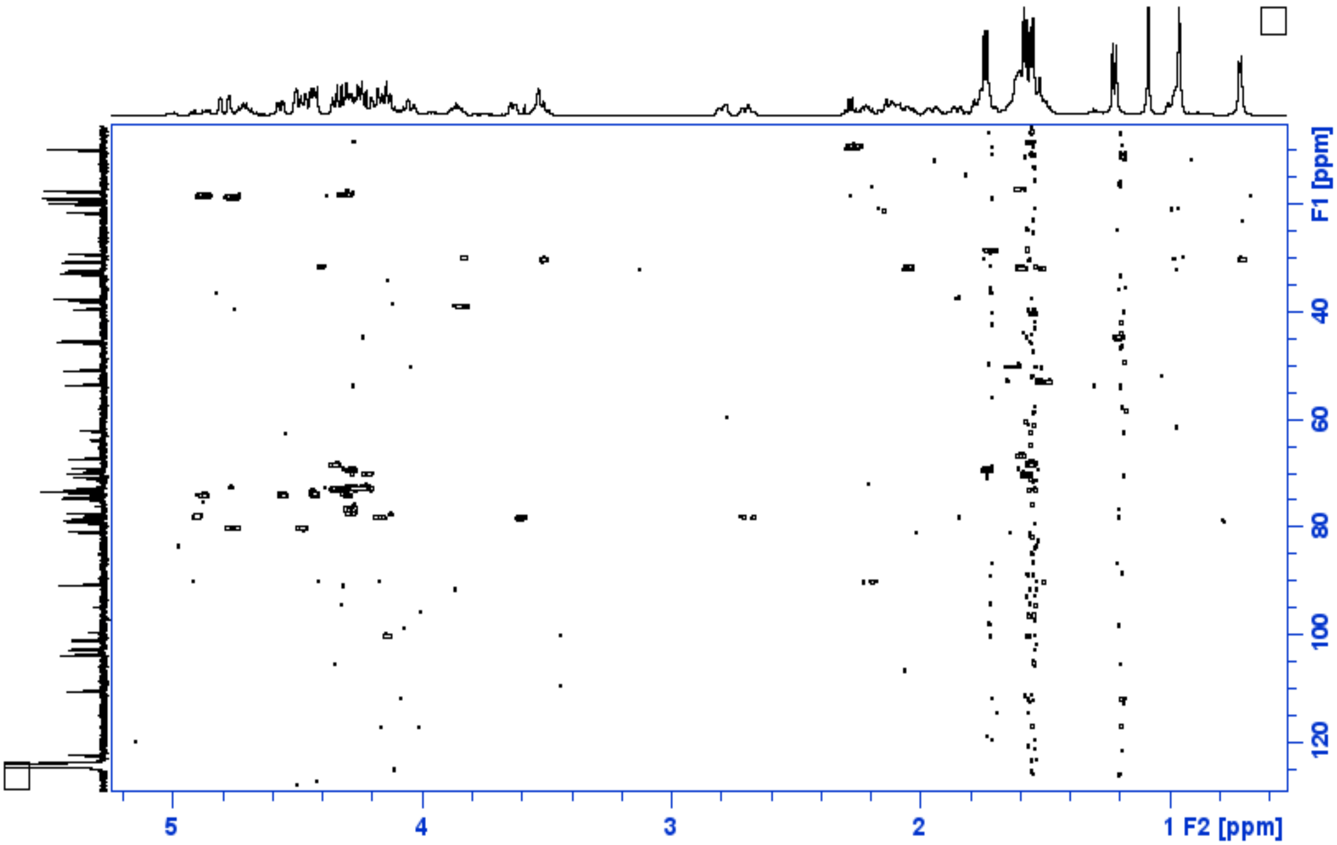


**Figure S15.** H2BC NMR (Py-d5, 50°C) spectra of pennogenin tetraglycoside

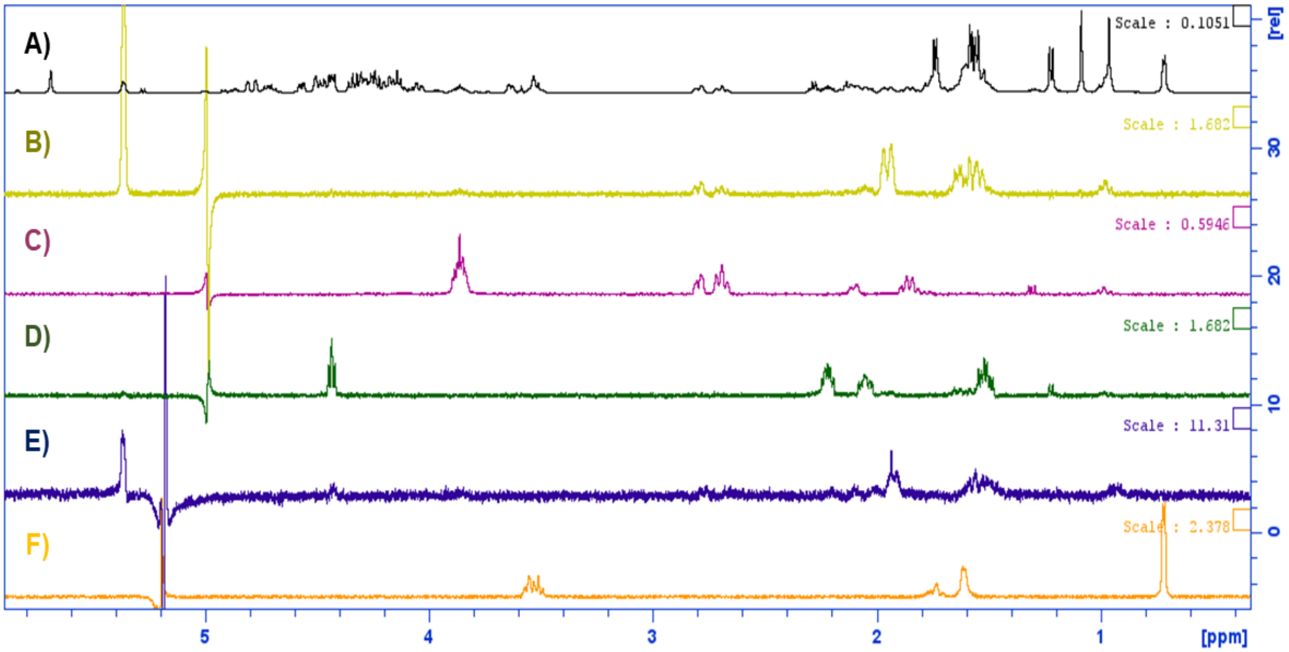


**Figure S16.** Stacked spectra of different TOCSY 1D at different chemical shifts of aglycone. A) $^{1}H$ NMR of pennogenin tetraglycoside B) Irradiation at 5.36 ppm C) Irradiation at 3.86 ppm D) Irradiation at 2.25 ppm E) Irradiation at 1.95 ppm F) Irradiation at 0.71 ppm

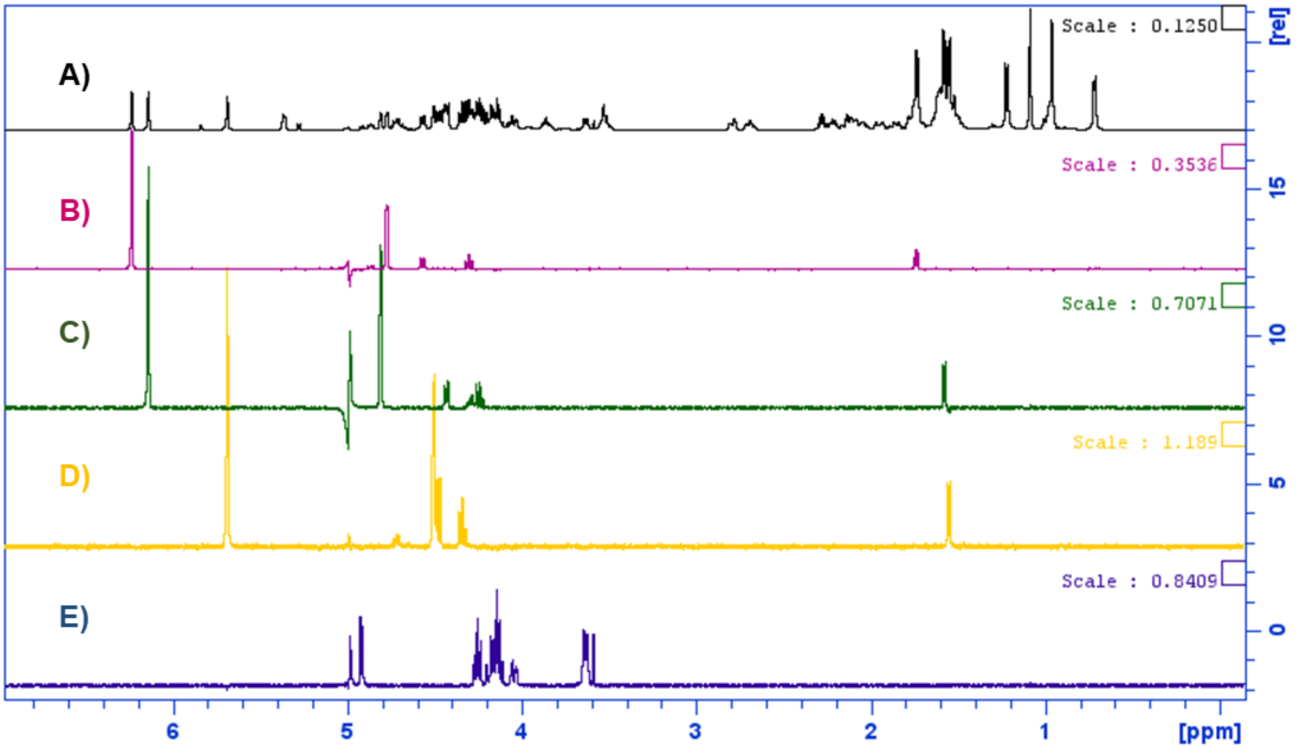


**Figure S17.** Stacked spectra of different TOCSY 1D at different chemical shifts of glycosidic portion. A) $^1H$ NMR of pennogenin tetraglycoside B) Irradiation at 6.23 ppm C) Irradiation at 6.14 ppm D) Irradiation at 5.69 ppm E) Irradiation at 4.92 ppm

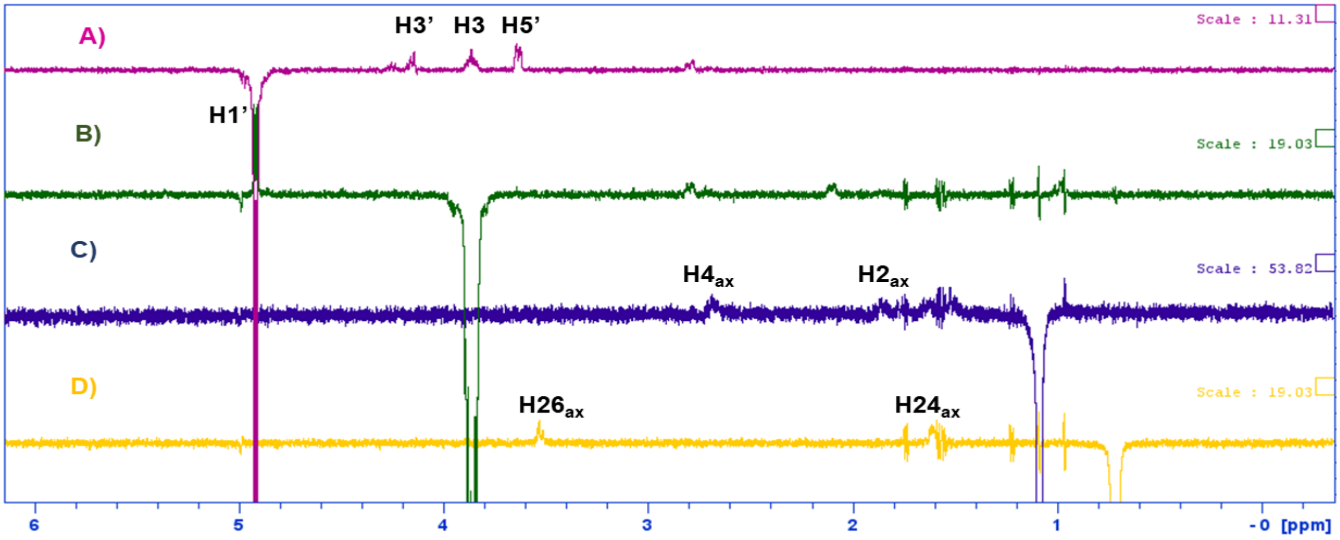


**Figure S18.** Stacked spectra of different ROESY 1D at different chemical shifts. A) Irradiation at 4.92 ppm proton H1′ B) Irradiation at 3.86 ppm proton H3 C) Irradiation at 1.08 ppm Methyl 19 D) Irradiation at 0.71 ppm Methyl 27

**Table S1.** Optimized dynamic multiple reaction monitoring method (dMRM) parameters for detection and quantification of Phenolic compounds

| Phenolic compound | Parent Ion (*m/z*) | Product Ion (*m/z*) | Retention Time (min) | Search Time (min) | Fragmentation Voltage (V) | Collision Voltage (V) | Polarity |
|---|---|---|---|---|---|---|---|
| Shikimic acid | 173.1 | 111.1 | 1.7 | 0.5 | 100 | 10 | - |
| Gallic acid | 169 | 125.2 | 3.8 | 0.5 | 100 | 10 | - |
| Protocatechuic acid | 153 | 109.1 | 6.4 | 0.25 | 100 | 10 | - |
| 4-hydroxybenzoic acid | 137.1 | 92.8 | 9.11 | 0.5 | 100 | 10 | - |
| Gentisic acid | 153 | 109 | 9.2 | | 100 | 10 | - |
| (-)-Epigallocatechin | 305.1 | 125 | 10.44 | 0.5 | 100 | 20 | - |
| 4-Hydroxyphenylacetic acid | 107.1 | 77 | 10.52 | 0.5 | 100 | 20 | - |
| (+)-Catechin | 291 | 138.9 | 11.2 | 0.5 | 100 | 10 | + |
| Vanillic acid | 169 | 93.03 | 11.81 | 0.5 | 100 | 10 | + |
| Scopolin | 355.1 | 193 | 11.87 | 0.5 | 100 | 20 | + |
| Chlorogenic acid | 355.1 | 163.03 | 12.12 | 0.3 | 100 | 10 | + |
| Caffeic acid | 181.04 | 163.03 | 12.24 | 0.5 | 100 | 10 | + |
| Procyanidin B2 | 577.1 | 425.1 | 13.69 | 0.5 | 100 | 10 | - |
| (-)-Epicatechin | 291 | 138.8 | 14.48 | 0.5 | 100 | 10 | + |
| Vanillin | 153 | 124.9 | 14.99 | 0.5 | 100 | 10 | + |
| Mangiferin | 423 | 302.28 | 15.18 | 0.5 | 100 | 10 | + |
| Keracyanin | 595.2 | 287.1 | 15.2 | 0.5 | 100 | 20 | + |
| 4-Coumaric acid | 165.05 | 147.04 | 16.22 | 0.25 | 100 | 10 | + |
| (-)-Gallocatechin gallate | 107 | 139 | 16.57 | 0.5 | 100 | 20 | + |
| Umbelliferone | 163 | 107 | 17.1 | 0.5 | 100 | 30 | + |
| Quercetin-3,4′-di-O-glucoside | 627 | 302.9 | 17.87 | 0.5 | 100 | 10 | + |
| Scopoletin | 193 | 133 | 18.44 | 0.5 | 100 | 10 | + |
| 3-Coumaric acid | 165.05 | 147.04 | 18.7 | 0.5 | 100 | 10 | + |
| Ferulic acid | 195.1 | 145.02 | 19.18 | 0.5 | 100 | 20 | + |
| Sinapic acid | 225.1 | 207.1 | 19.57 | 0.5 | 100 | 10 | + |
| (-)-Epicatechin gallate | 443.1 | 123 | 19.91 | 0.5 | 100 | 10 | + |
| Myricitrin | 465 | 318.9 | 20.25 | 0.5 | 100 | 10 | + |
| Ellagic acid | 301 | 145 | 20.36 | 0.5 | 100 | 40 | - |
| Quercetin-3-D-galactoside | 465 | 302.9 | 20.6 | 0.5 | 100 | 10 | - |
| Quercetin-3-glucoside | 465 | 303 | 21.9 | 0.5 | 100 | 10 | + |
| Luteolin-7-O-glucoside | 449 | 287 | 21.4 | 0.6 | 100 | 10 | + |
| *p*-Anisic acid | 153.1 | 109 | 22.21 | 0.5 | 120 | 5 | + |
| Penta-O-galloyl-β-D-glucose | 771.1 | 153 | 22.27 | 0.5 | 100 | 20 | + |
| 2,4-Dimethoxy-6-methylbenzoic acid | 197 | 179 | 23.43 | 0.5 | 80 | 5 | + |
| Kaempferol-3-O-glucoside | 449 | 286.9 | 23.64 | 0.5 | 100 | 10 | + |
| Naringin | 273 | 153 | 23.64 | 0.5 | 120 | 10 | + |

| | | | | | | | |
|---|---|---|---|---|---|---|---|
| Quercitrin | 449.1 | 303.1 | 23.71 | 0.5 | 100 | 10 | + |
| Hesperidin | 609.1 | 301.1 | 24.55 | 0.5 | 100 | 20 | - |
| Myricetin | 317 | 179 | 24.64 | 0.5 | 100 | 10 | - |
| Rosmarinic acid | 361.1 | 163 | 24.85 | 0.5 | 100 | 10 | + |
| Phloridzin | 435 | 272.9 | 25.21 | 0.5 | 100 | 10 | - |
| trans-Resveratrol | 229.1 | 135 | 25.86 | 0.5 | 100 | 10 | + |
| trans-Cinnamic acid | 149.1 | 131 | 28.5 | 0.5 | 100 | 10 | + |
| Cirsimarin | 477 | 314.9 | 29.4 | 0.5 | 100 | 10 | + |
| Quercetin | 302.9 | 153.1 | 29.7 | 0.5 | 100 | 35 | + |
| Psoralen | 187 | 131.1 | 31.4 | 0.5 | 100 | 20 | + |
| Angelicin | 187 | 131.1 | 32.74 | 0.5 | 100 | 20 | + |
| Naringenin | 271 | 151 | 32.83 | 0.5 | 100 | 10 | - |
| Apigenin | 271 | 153 | 34.1 | 0.5 | 100 | 30 | + |
| Kaempferol | 287.1 | 153 | 34.79 | 0.5 | 100 | 30 | + |
| Hesperetin | 303.1 | 177.1 | 34.8 | 0.5 | 100 | 20 | + |
| Citropten | 207.06 | 192.04 | 36.11 | 0.5 | 100 | 20 | + |
| Nordihydroguaiaretic acid | 303 | 193.1 | 42.36 | 0.5 | 100 | 10 | + |
| Chrysin | 255.1 | 153 | 43.13 | 0.5 | 100 | 40 | + |
| Kaempferide | 301 | 258.2 | 43.5 | 0.5 | 100 | 20 | + |
| Emodin | 269 | 225 | 44.2 | 0.5 | 150 | 20 | - |
| Chrysophanol | 255.1 | 153 | 45.3 | 0.5 | 100 | 40 | + |
